# Effects of whole-body vibration training on physical function, activities of daily living, and quality of life in patients with stroke: a systematic review and meta-analysis

**DOI:** 10.3389/fphys.2024.1295776

**Published:** 2024-01-23

**Authors:** Duchun Zeng, Kun Zhao, Wei Lei, Yanmei Yu, Weili Li, Yurou Kong, Junmei Lai, Fenghao Ma, Xiangming Ye, Xiaofeng Zhang

**Affiliations:** ^1^ Center for Rehabilitation Medicine, Rehabilitation & Sports Medicine Research Institute of Zhejiang Province, Department of Rehabilitation Medicine, Zhejiang Provincial People’s Hospital (Affiliated People’s Hospital), Hangzhou Medical College, Hangzhou, China; ^2^ Graduate School, Zhejiang Chinese Medical University, Hangzhou, China; ^3^ Department of Physiotherapy, Shanghai Sunshine Rehabilitation Center, Tongji University School of Medicine, Shanghai, China

**Keywords:** whole-body vibration, stroke, physical function, activities of daily living, meta-analysis

## Abstract

**Purpose:** This systematic review and meta-analysis aimed to evaluate the efficacy of whole-body vibration training (WBVT) in patients with stroke, specifically focusing on its effects on physical function, activities of daily living (ADL), and quality of life (QOL). Additionally, potential moderators influencing WBVT outcomes were explored.

**Methods:** We conducted a systematic search of PubMed, Embase, and Cochrane Library from inception to September 2022. Eligible studies were randomized controlled trials employing WBVT in patients with stroke. Two investigators independently extracted the data and calculated the standardized mean difference (SMD) using random-effect models.

**Results:** Twenty-five studies involving 991 patients were included in this meta-analysis. WBVT demonstrated significant reductions in spasticity (SMD = −0.33, 95% CI = −0.61 to −0.06, *p* = 0.02), improvements in motor function (SMD = 0.39, 95% CI = 0.16 to 0.61, *p* < 0.01), and enhancements in balance function (SMD = 0.28, 95% CI = 0.09 to 0.47, *p* < 0.01) in patients with stroke. However, no significant effects were observed for gait (SMD = −0.23, 95% CI = −0.50 to 0.04, *p* = 0.10), ADL (SMD = −0.01, 95% CI = −0.46 to 0.44, *p* = 0.97), or QOL (SMD = 0.12, 95% CI = −0.30 to 0.53, *p* = 0.59). Subgroup analyses revealed that variable frequency vibration and side-alternating vibration exhibited significant efficacy in reducing spasticity and improving motor and balance functions, while fixed frequency vibration and vertical vibration did not yield significant therapeutic benefits in these domains.

**Conclusion:** Our findings indicate that WBVT may serve as a viable adjunct therapy for stroke patients to alleviate spasticity and enhance motor and balance functions. Variable frequency and side-alternating vibration appear to be crucial factors influencing the therapeutic effects of WBVT on these dysfunctions. Nonetheless, WBVT did not show significant effects on gait, ADL, or QOL in stroke patients.

**Systematic Review Registration**: https://www.crd.york.ac.uk/PROSPERO/, identifier (CRD42022384319)

## 1 Introduction

Stroke has emerged as a global public health challenge and social issue. Despite variations in the epidemiological statistics of stroke across different regions of the world, it is still characterized by high morbidity, mortality, and disability rates (Feigin et al., 2022). Patients with stroke, in many cases, struggle with the long-term functional deficits, including spasticity, motor impairment, balance disorder, walking dysfunction, and limited activities of daily living (ADL), which can impact their quality of life (QOL) and place a heavy burden on their families and society as a whole (Langhorne et al., 2009; Morris et al., 2017; Alawieh et al., 2018). Given the complex and multifactorial nature of post-stroke functional recovery, an increasing number of trials have explored various rehabilitation strategies to identify potential benefits (Morris et al., 2017). Whole-body vibration training (WBVT) is a non-pharmaceutical intervention that involves placing individuals on a vibration platform in specific postures, such as sitting or standing, to transmit vibration stimulation throughout the body and elicit physiological effects (Sadeghi and Sawatzky, 2014). Numerous studies have demonstrated the effectiveness of WBVT in enhancing muscle strength, physiological parameters, flexibility, balance, and coordination across diverse populations, including athletes, older adults, individuals with metabolic syndrome, and patients with neurological conditions such as spinal cord injury, Parkinson’s disease, cerebral palsy, and multiple sclerosis (Gusso et al., 2016; Bemben et al., 2018; In et al., 2018; Sá-Caputo et al., 2018; Sierra-Guzmán et al., 2018; Sá-Caputo et al., 2019; Andreu et al., 2020; Zhao et al., 2023). Furthermore, WBVT has shown promise in improving spasticity, gait deficits, poor balance, and sensory abnormalities in patients with musculoskeletal conditions such as knee osteoarthritis (Simão et al., 2019). With the advancement of WBVT and its wide application in clinical settings, its potential role in stroke rehabilitation has been gradually valued (Liao et al., 2014; Yang and Butler, 2020).

In recent years, several studies have investigated the effects of WBVT on physical function in patients with stroke (Liao et al., 2014; Lu et al., 2015; Yang et al., 2015; Park et al., 2018; Yang and Butler, 2020; Yang et al., 2022). However, these studies have not yielded consistent results, with certain studies indicating significant enhancements in physical function (Yang and Butler, 2020; Yang et al., 2022), while others have failed to find sufficient evidence to support clinical use of WBVT on physical function (Lu et al., 2015; Yang et al., 2015; Park et al., 2018). Disparities among these studies could be attributed to differences in study design, sample size, intervention protocols, and outcome measures. Consequently, the overall efficacy of WBVT for physical function remains unclear. Notably, previous meta-analyses have attempted to reveal the efficacy of WBVT in patients with stroke, but these studies have focused solely on lower extremity function, such as fall risk, balance, and gait (Lu et al., 2015; Yang et al., 2015; Park et al., 2018; Yang and Butler, 2020; Yang et al., 2022). In addition to physical function, it may also be of practical value to further explore the effects of WBVT on ADL and QOL in patients with stroke. Additionally, it should be noted that previous meta-analyses included non-randomized controlled trials (RCTs) (Park et al., 2018; Yang and Butler, 2020), which may lead to the bias of research conclusions. Furthermore, most of the previous meta-analyses of WBVT on different functional outcomes included small sample sizes (Lu et al., 2015; Yang et al., 2015; Park et al., 2018; Yang and Butler, 2020; Yang et al., 2022), and some new relevant RCTs have not been included. Finally, the selection of WBVT parameters is a crucial factor for effective treatment in clinical practice. However, there are currently no guidelines regarding the appropriate parameters for WBVT in patients with stroke, and previous meta-analyses did not pay enough attention to the efficacy of optimal WBVT parameters.

Therefore, the aim of this systematic review and meta-analysis is to synthesize the available evidence on the effects of WBVT on physical function, ADL, and QOL in patients with stroke. Specifically, we will 1) determine the overall effect of WBVT on physical function, ADL, and QOL in patients with stroke; 2) explore the potential moderators of the effects of WBVT, such as vibration direction, frequency mode.

## 2 Materials and methods

This meta-analysis was reported in accordance with the Preferred Reporting Items for Systematic Reviews and Meta-Analyses (PRISMA) (Page et al., 2021). The protocol was pre-registered on PROSPERO (CRD42022384319).

### 2.1 Search strategy

We searched PubMed, Embase, and Cochrane Library databases from inception to September 2022. All RCTs on WBVT in patients with stroke were searched. Search strategies for different databases are presented in Supplementary Table S1. The relevant reference lists in previous studies were checked to avoid possible omissions.

### 2.2 Eligibility criteria

Two evaluators independently screened articles for eligibility based on the following criteria: 1) Participants: patients with stroke. 2) Interventions: WBVT defined in the experimental group. 3) Comparisons: routine rehabilitation (no WBVT or sham WBVT). 4) Physical function included spasticity, motor function, balance function, and gait performance; spasticity was measured by the Modified Ashworth Scale (MAS); motor function was assessed by the Fugl-Meyer Assessment (FMA), Manual Function Test (MFT), Rivermead Mobility Index (RMI), or Wolf Motor Function Test (WMFT); balance function was estimated by the Berg Balance Scale (BBS), Korea Berg Balance Scale (K-BBS), Activities Specific Balance Confidence (ABC), Tinetti Performance Oriented Mobility Assessment (TPOMA), Functional Reach Test (FRT), Modified Functional Reach Test (MFRT), or Mini Balance Evaluation Systems Test (Mini-BESTest); gait performance was measured by the Timed-Up-and-Go test (TUGT), 10-m Walk Test (10MWT), 6-Minute Walk Test (6MWT), Functional Ambulatory Category (FAC), or Tinetti Performance Oriented Mobility Assessment (TPOMA). 5) ADL was evaluated by the Barthel Index (BI), Korea modified Barthel Index (K-MBI), Functional Independence Measure (FIM), Frenchay Activity Index (FAI). 6) QOL was measured by the Stroke Impact Scale (SIS), Short-Form 12 Health Survey (SF-12). 7) Study design: RCTs. 8) Language: articles were published in English. Exclusion criteria included: 1) Unextractable data. 2) Abstracts or congress conference papers.

### 2.3 Data extraction

Two evaluators independently designed the electronic information form and extracted the following variables: 1) Study characteristics. 2) Participants characteristics. 3) Characteristics of WBVT. 4) Outcome data (the mean and standard deviation of scale scores). Non-compliant data formats were converted using the Cochrane Handbook’s recommended method. For studies with multiple WBVT groups with different frequencies, these groups were considered independent trials. If a multi-arm trial included WBVT with another intervention, we excluded the additional experimental group from analysis. When conducting an overall combined analysis of a functional outcome, if a study used multiple similar assessment scales, we selected the most commonly used, widely accepted, and reliably validated scale for inclusion in the overall analysis. This approach increased the comparability and consistency of the findings. Discrepancies between evaluators were resolved by discussion or input from a third evaluator.

### 2.4 Risk of bias

The Cochrane Collaboration tool was developed for evaluating the bias risk of each eligible study (Sterne et al., 2019). Two evaluators independently used this tool to estimate the risk of selection bias, performance bias, detection bias, attrition bias, reporting bias, and other biases in each study.

### 2.5 Data analysis

The data were analyzed using Stata (version 17.0, Stata Corp, United States). The effect sizes of WBVT on functional outcomes were demonstrated using standardized mean difference (SMD) and 95% confidence interval (CI). The random effects model was applied to measure mixed effects, with *p* < 0.05 considered statistically significant. Heterogeneity was assessed using *I* ^2^ statistics, with *I* ^2^ > 50% indicating significant heterogeneity among studies. Publication bias was evaluated through funnel plots and Egger’s test, with asymmetric or a probability value of *p* < 0.05 indicating significant publication bias. Sensitivity analysis was performed by removing each study individually to assess the reliability of results. Subgroup analyses were mainly performed to explore the factors (frequency mode and vibration direction) influencing the effects of WBVT on physical function.

## 3 Results

### 3.1 Search results

**FIGURE 1:**
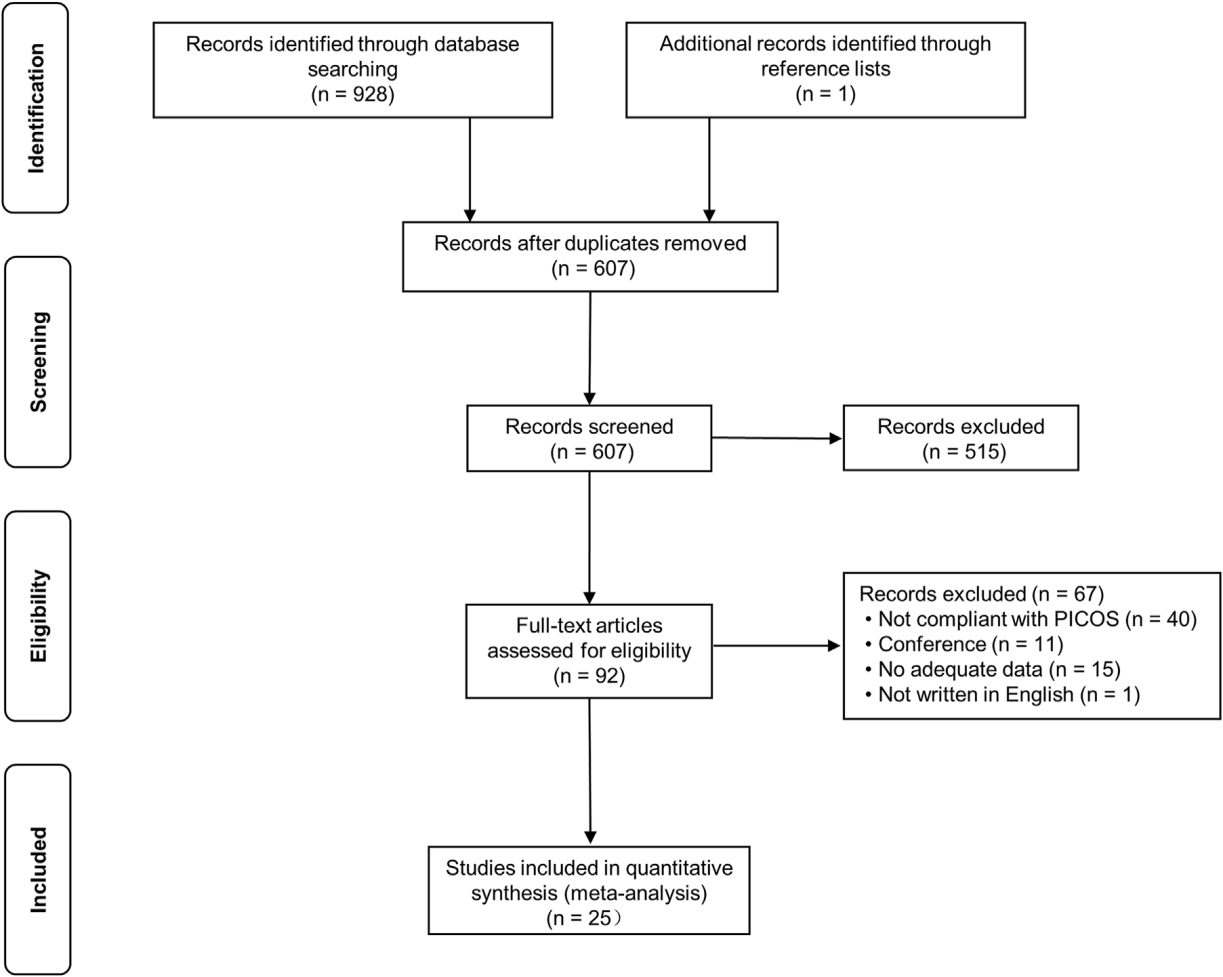
PRISMA flow diagram depicting the study process. PICOS: participants, interventions, comparisons, outcome, and study design.

The PRISMA screening process was illustrated in Figure 1. A total of 928 records were retrieved from the database, and one record was identified from the references of previous studies. After removing duplicates, 607 remained. Following the scanning of title and abstract, 515 records were excluded, leaving 92 studies for full-text review. Ultimately, 25 articles met the conditions of quantitative meta-analysis.

### 3.2 Study characteristics

The characteristics of the 25 selected studies are presented in Table 1. The studies were conducted in different regions, including 17 in Asia (Chan et al., 2012; Lau et al., 2012; Nagino et al., 2013; Choi et al., 2014; Guo et al., 2015; Lee, 2015; Lee et al., 2016a; Choi et al., 2016; Liao et al., 2016; Choi et al., 2017; Lee et al., 2017; Hwang, 2018; Ahn et al., 2019; Dang and Chen, 2019; Lee et al., 2020; Kim and Lee, 2021; Wei and Cai, 2022), 5 in Europe (van Nes et al., 2006; Merkert et al., 2011; Brogårdh et al., 2012; Marín et al., 2013; Tankisheva et al., 2014), 1 in America (Silva et al., 2014), and 2 in Turkey (Alp et al., 2018; Sade et al., 2020). A total of 991 individuals were enrolled, with 6 studies not reporting specific stroke subtypes (Merkert et al., 2011; Choi et al., 2014; Choi et al., 2016; Choi et al., 2017; Alp et al., 2018; Dang and Chen, 2019), 3 articles not providing the side of lesion (Merkert et al., 2011; Guo et al., 2015; Ahn et al., 2019), and 3 articles not providing disease duration (Merkert et al., 2011; Choi et al., 2016; Lee et al., 2020). Most studies reported subacute and chronic stages. Frequency mode of WBVT was categorized into fixed frequency (van Nes et al., 2006; Brogårdh et al., 2012; Chan et al., 2012; Nagino et al., 2013; Silva et al., 2014; Choi et al., 2016; Liao et al., 2016; Lee et al., 2017; Alp et al., 2018; Lee et al., 2020; Kim and Lee, 2021; Wei and Cai, 2022) and variable frequency (Merkert et al., 2011; Lau et al., 2012; Marín et al., 2013; Choi et al., 2014; Tankisheva et al., 2014; Guo et al., 2015; Lee, 2015; Lee et al., 2016a; Choi et al., 2017; Hwang, 2018; Ahn et al., 2019; Dang and Chen, 2019; Sade et al., 2020). According to the vibration direction, WBVT can be divided into side-alternating (van Nes et al., 2006; Marín et al., 2013; Nagino et al., 2013; Choi et al., 2014; Lee, 2015; Lee et al., 2016a; Choi et al., 2016; Choi et al., 2017; Hwang, 2018; Ahn et al., 2019; Dang and Chen, 2019; Lee et al., 2020; Wei and Cai, 2022) and vertical vibration (Merkert et al., 2011; Brogårdh et al., 2012; Chan et al., 2012; Lau et al., 2012; Tankisheva et al., 2014; Guo et al., 2015; Liao et al., 2016; Lee et al., 2017; Alp et al., 2018; Sade et al., 2020; Kim and Lee, 2021).

**TABLE 1:** Characteristics of the included studies.

Study	Country	Sample size	Sex (M/F)	Age mean (SD), year	Side of lesion (L/R)	Type of stroke (I/H)	Disease duration mean (SD)	Frequency(Hz)	Frequency mode	Amplitude	Vibration direction	Frequency of sessions, duration of program	Control intervention	Main outcomes
Ahn et al. (2019)	Korea	TG = 30	18/12	58.7 (7.1)	NR	15/15	95.7 (31.5) days	4–19	Variable frequency	NR	Side-alternating	5 days/week, 4 weeks	WBVT	MFT
		CG = 30	17/16	60.7 (5.9)	NR	14/16	77.3 (30.3) months						No WBVT	
Alp et al. (2018)	Turkey	TG = 10	10/0	61.20 (11.04)	4/6	NR	1.5 (0.75) years	40	Fixed frequency	4 mm	Vertical	3 days/week, 4 weeks	WBVT	MAS, FIM, 10MWT
		CG = 11	9/2	62.91 (8.15)	8/3	NR	2 (1.13) years						Sham WBVT	
Brogårdh et al. (2012)	Sweden	TG = 16	13/3	61.3 (8.5)	7/9	14/2	37.4 (31.8) months	25	Fixed frequency	3.75 mm	Vertical	2 days/week, 6 weeks	WBVT	MAS, BBS, TUGT, 6MWT, SIS
		CG = 15	12/3	63.9 (5.8)	8/7	13/2	33.1 (29.2) months	25		0.2 mm			Sham WBVT	
Chan et al. (2012)	China	TG = 15	10/5	56.07 (11.04)	12/3	10/5	30.40 (25.80) months	12	Fixed frequency	4 mm	Vertical	1 session	WBVT	MAS, TUGT, 10MWT
		CG = 15	11/4	54.93 (7.45)	7/8	5/10	38.87 (38.22) months						Sham WBVT	
Choi et al. (2016)	Korea	TG = 11	8/3	50.9 (8.2)	7/4	NR	6 months or longer	25	Fixed frequency	5 mm	Side-alternating	5 days/week, 4 weeks	WBVT	FRT, TUGT
		CG = 11	7/4	52.2 (12.3)	4/7	NR	6 months or longer						No WBVT	
Choi et al. (2014)	Korea	TG = 15	9/6	62.8 (9.0)	6/9	NR	13.0 (5.4) months	15–22	Variable frequency	0–5.8 mm	Side-alternating	5 days/week, 4 weeks	WBVT	MFRT
		CG = 15	7/8	65.1 (15.7)	5/10	NR	12.6 (5.7) months						No WBVT	
Choi et al. (2017)	Korea	TG = 15	8/7	51.93 (8.35)	8/7	NR	25.13 (9.25) months	20–30	Variable frequency	3 mm	Side-alternating	3 days/week, 6 weeks	WBVT	6MWT
		CG = 15	11/4	53.67 (7.38)	5/10	NR	22.53 (10.27) months						Sham WBVT	
Dang and Chen (2019)	China	TG = 32	20/12	57.8 (8.5)	14/18	NR	6.6 (3.6) months	5–15	Variable frequency	1–6 mm	Side-alternating	5 days/week, 4 weeks	WBVT	FMA, WMFT, MAS
		CG = 30	19/11	60.0 (9.0)	12/18	NR	8.1 (3.6) months						No WBVT	
Guo et al. (2015)	China	TG = 15	NR	53.8 (6.0)	NR	10/5	66.9 (42.9) months	6–10	Variable frequency	4 mm	Vertical	5 days/week, 8 weeks	WBVT	FMA, 10MWT
		CG = 15	NR	54.3 (6.8)	NR	12/3	59.4 (61.4) months						Sham WBVT	
Hwang (2018)	Korea	TG = 9	6/3	66.0 (14.82)	4/5	7/2	21.0 (11.85) days	20–30	Variable frequency	2–3 mm	Side-alternating	5 days/week, 4 weeks	WBVT	K-BBS, MMT, MAS, FAC, K-MBI
		CG = 9	4/5	71.0 (5.18)	5/4	8/1	21.0 (8.89) days						Sham WBVT	
Kim and Lee (2021)	Korea	TG = 20	8/12	57.20 (11.00)	9/11	15/5	31.60 (15.18) days	16	Fixed frequency	NR	Vertical	5 days/week, 2 weeks	WBVT	BBS, 10MWT, TUGT, FAC
		CG = 18	7/11	55.70 (10.40)	11/7	10/8	28.00 (8.72) days						No WBVT	
Lau et al. (2012)	China	TG = 41	26/15	57.3 (11.3)	21/20	20/21	4.6 (3.5) years	20–30	Variable frequency	0.44–0.60 mm	Vertical	3 days/week, 8 weeks	WBVT	BBS, 6MWT, 10MWT, ABC
		CG = 41	32/9	57.4 (11.1)	27/14	21/20	5.3 (4.2) years						Sham WBVT	
Lee (2015)	Korea	TG = 12	8/4	59.3 (13.2)	5/7	7/5	19.0 (9.1) months	1–3	Variable frequency	30 mm	Side-alternating	3 days/week, 6 weeks	WBVT	FMA, BBS, TUGT
		CG = 9	6/3	56.0 (9.1)	6/3	7/2	18.0 (10.9) months						No WBVT	
Lee et al. (2016)	Korea	TG = 15	5/10	59.20 (7.72)	9/6	7/8	7.99 (4.23) months	5–15	Variable frequency	1–6 mm	Side-alternating	3 days/week, 4 weeks	WBVT	FMA, WMFT, MAS
		CG = 15	9/6	60.24 (6.73)	5/10	8/7	6.71 (3.85) months						No WBVT	
Lee et al. (2017)	Korea	TG = 15	8/7	59.1 (16.9)	10/5	8/7	18.4 (6.9) days	40	Fixed frequency	NR	Vertical	5 days/week, 2 weeks	WBVT	K-MBI, FAC, BBS, TIS
		CG = 15	7/8	64.4 (14.8)	9/6	9/6	25.3 (12.2) days						No WBVT	
Lee et al. (2020)	Korea	TG = 15	12/3	58.73 (9.09)	9/6	10/5	6 months or longer	20	Fixed frequency	4 mm	Side-alternating	1session	WBVT	10MWT, TUGT, FMA, TPOMA
		CG = 14	11/3	53.42 (9.55)	11/3	10/4	6 months or longer						Sham WBVT	
Liao et al. (2016) A	China	TG = 28	20/8	60.8 (8.3)	20/8	16/12	8.5 (5.2) months	20	Fixed frequency	1 mm	Vertical	3 days/week, 10 weeks	WBVT	MAS, Mini-BESTest, TUGT, 6MWT, ABC, FAI, SF12
		CG = 28	24/4	59.8 (9.1)	12/16	17/11	9.0 (4.6) months						Sham WBVT	
Liao et al. (2016) B	China	TG = 28	18/10	62.9 (10.2)	19/9	16/12	8.1 (4.2) months	30	Fixed frequency	1 mm	Vertical	3 days/week, 10 weeks	WBVT	MAS, Mini-BESTest, TUGT, 6MWT, ABC, FAI, SF12
		CG = 28	24/4	59.8 (9.1)	12/16	17/11	9.0 (4.6) months						Sham WBVT	
Marín et al. (2013)	Spain	TG = 11	6/5	62.3 (10.6)	6/5	10/1	4.3 (2.0) years	5–21	Variable frequency	2–3 mm	Side-alternating	1 day/week (1–7 weeks),2 days/week (8–12 weeks), 12w	WBVT	BBS
		CG = 9	5/4	64.4 (7.6)	4/5	7/2	4.3 (3.0) years						Sham WBVT	
Merkert et al. (2011)	Germany	TG = 33	11/22	74.5 (8.6)	NR	NR	NR	20–45	Variable frequency	NR	Vertical	15 days	WBVT	BBS, TUGT, BI
		CG = 33	11/22	74.5 (8.6)	NR	NR	NR						No WBVT	
Nagino et al. (2013)	Japan	TG = 15	8/7	68.9 (11.6)	10/5	9/6	11.3 (1.2) months	20	Fixed frequency	3 mm	Side-alternating	3 days/week, 4 weeks	WBVT	BBS, FMA, FRT
		CG = 15	10/5	70.2 (7.7)	7/8	7/8	8.7 (1.1) months						No WBVT	
Sade et al. (2020)	Turkey	TG = 26	12/14	46.8 (15.0)	17/9	21/5	34.5 (25) months	35–40	Variable frequency	2 mm	Vertical	5 days/week, 3 weeks	WBVT	BBS, TUGT
		CG = 17	8/9	51.6 (10)	11/6	11/6	35.5 (20) months						No WBVT	
Silva et al. (2014)	Brazil	TG = 28	19/9	60.75 (11.80)	11/17	25/3	40.85 (68.76) months	50	Fixed frequency	2 mm	NR	1session	WBVT	6MWT, TUGT
		CG = 10	8/2	58.1 (8.14)	3/7	8/2	39.61 (63.55) months						Sham WBVT	
Tankisheva et al. (2014)	Belgium	TG = 7	4/3	57.4 (13)	4/3	6/1	7.71 (8.6) years	35/40	Variable frequency	1.7/2.5 mm	Vertical	3 days/week, 6 weeks	WBVT	MAS
		CG = 8	4/4	65.3 (3.7)	4/4	5/3	5.28 (3.6) years						No WBVT	
van Nes et al. (2006)	Netherlands	TG = 27	16/11	59.7 (12.3)	14/13	16/11	38.9 (9.2) days	30	Fixed frequency	3 mm	Side-alternating	5 days/week, 6 weeks	WBVT	BBS, RMI, BI, FAC, MI
		CG = 26	14/12	62.6 (7.6)	11/15	22/4	34.2 (11.1) days						No WBVT	
Wei and Cai (2022) A	China	TG = 26	23/3	72.42 (5.89)	14/12	15/11	33.65 (15.75) months	13	Fixed frequency	NR	Side-alternating	5 days/week, 2 weeks	WBVT	BBS, 10MWT, TUGT
		CG = 26	21/5	71.85 (6.03)	10/16	15/11	31.23 (19.33) months						Sham WBVT	
Wei and Cai (2022) B	China	TG = 26	21/5	70.19 (5.07)	13/13	17/9	36.69 (20.32) months	26	Fixed frequency	NR	Side-alternating	6 days/week, 2 weeks	WBVT	BBS, 10MWT, TUGT
		CG = 26	21/5	71.85 (6.03)	10/16	15/11	31.23 (19.33) months						Sham WBVT	

Note: ABC, activities specific balance confidence; BBS, berg balance scale; BI, barthel index; CG, control group; FAC, functional ambulatory category; FAI, frenchay activity index; FIM, functional independence measure; FMA, Fugl-Meyer Assessment; FRT, functional reach test; K-BBS, korea berg balance scale; K-MBI, korea modified barthel index; I/H, Infarction/Hemorrhage; L/R, Left/Right; M/F, Male/Female; MAS, modified ashworth scale; MFRT, modified functional reach test; MFT, manual function test; Mini-BESTest, Mini Balance Evaluation Systems Test; NR, not report; RMI, rivermead mobility index; SD, standard deviation; SF-12, Short-Form 12 Health Survey; SIS, stroke impact scale; TG, treatment group; TPOMA, tinetti performance oriented mobility assessment; TUGT, Timed-Up-and-Go test; WMFT, wolf motor function test; 10MWT, 10-m Walk Test; 6MWT, 6-Minute Walk Test.

### 3.3 Quality appraisal of literature

**FIGURE 2:**
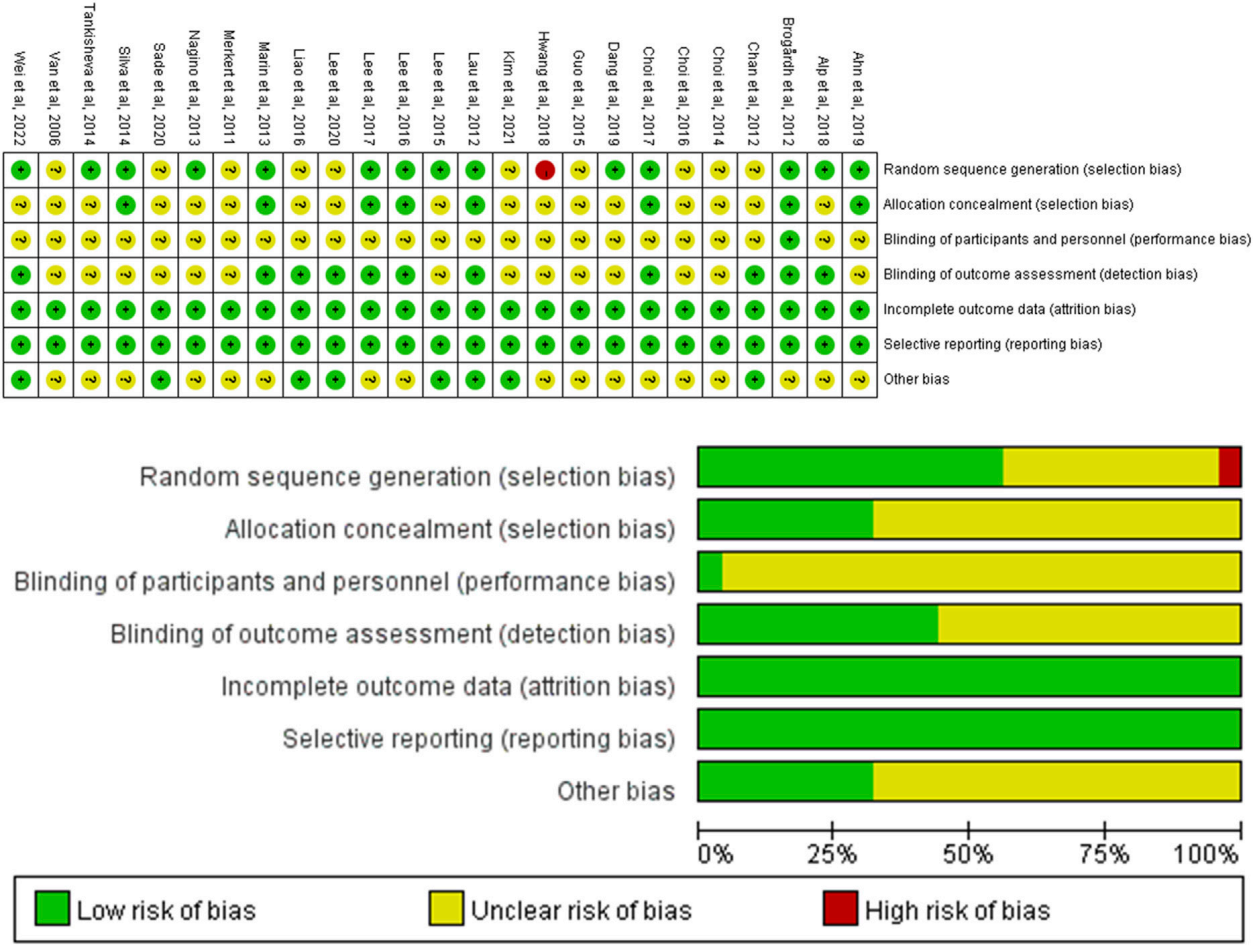
Bias risk assessment for included articles.

The assessment of bias risk in the included studies is presented in Figure 2. For the random sequence generation method, 14 studies were rated as low risk, and 1 study were rated as high risk. Eight studies used allocation concealment. The risk of blinding in participants was unclear in most studies. Eleven studies implemented a blind method for outcome evaluators and all studies reported a low risk of incomplete outcome data.

### 3.4 Meta-analysis

#### 3.4.1 Effects of WBVT on physical function

**FIGURE 3:**
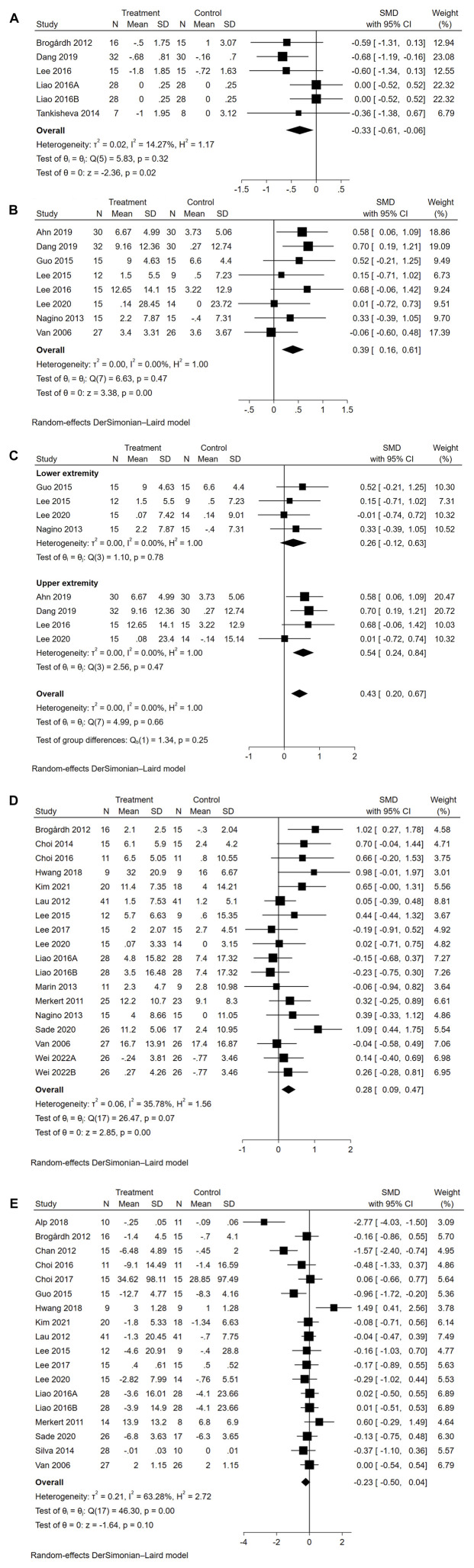
(Continued).

Five studies reported data on spasticity (Figure 3A). The overall effect size of WBVT on MAS was significant (SMD = −0.33, 95% CI = −0.61 to −0.06, *p* = 0.02), indicating that WBVT had an anti-spasticity effect compared to the control group.

Eight studies reported data on motor function. As illustrated in Figure 3B, the overall effect size of WBVT on motor function was significant (SMD = 0.39, 95% CI = 0.16 to 0.61, *p* < 0.01), indicating a potential effect size in favor of the WBVT group. Specifically, when examining extremity-specific motor function (Figure 3C), a significant improvement was observed in upper extremity motor function (SMD = 0.54, 95% CI = 0.24 to 0.84, *p* < 0.01). However, no significant improvement was found in lower extremity motor function (SMD = 0.26, 95% CI = −0.12 to 0.63, *p* = 0.18). Subgroup analyses were performed based on motor function scales, including the FMA, MFT, RMI and WMFT (Supplementary Figure S1). The results showed significant effect sizes for the FMA (SMD = 0.45, 95% CI = 0.17 to 0.73, *p* < 0.01) and MFT (SMD = 0.58, 95% CI = 0.06 to 1.09, *p* < 0.01), while nonsignificant effect sizes were found for the RMI (SMD = −0.06, 95% CI = −0.60 to 0.48) and WMFT (SMD = 0.16, 95% CI = −0.25 to 0.57).

Sixteen studies reported data on balance function. As seen in Figure 3D, the overall effect size of WBVT on balance was significant (SMD = 0.28, 95% CI = 0.09 to 0.47, *p* < 0.01), indicating a potential minor effect size in favor of the WBVT. Subgroup analyses based on the balanced function scales (Supplementary Figure S2) also revealed significant effect sizes for the BBS (SMD = 0.31, 95% CI = 0.09 to 0.53, *p* < 0.01) and FRT (SMD = 0.63, 95% CI = 0.07 to 1.19, *p* < 0.01). However, the other five balance scales, namely, the ABC (SMD = −0.03, 95% CI = −0.31 to 0.26), K-BBS (SMD = 0.98, 95% CI = −0.01 to 1.97), MFRT (SMD = 0.70, 95% CI = −0.04 to 1.44), Mini-BESTest (SMD = −0.24, 95% CI = −0.61 to 0.14), and TPOMA (SMD = 0.02, 95% CI = −0.71 to 0.75), did not show significant effect sizes.

Seventeen studies reported data on gait. As seen in Figure 3E, the overall effect size of WBVT on gait was insignificant (SMD = −0.23, 95% CI = −0.50 to 0.04, *p* = 0.10), indicating insufficient evidence to determine the efficacy of WBVT on gait. Subgroup analyses were conducted based on different gait scales (Supplementary Figure S3), the effect size of the 10MWT was significant (SMD = −0.73, 95% CI = −1.29 to −0.17, *p* < 0.01), indicating a slight improvement in gait. However, no statistically significant improvements were observed in the other four scales, including the 6MWT (SMD = 0.09, 95% CI = −0.14 to 0.33), FAC (SMD = 0.49, 95% CI = −0.20 to 1.18), TPOMA (SMD = 0.03, 95% CI = −0.70 to 0.76) and TUGT (SMD = −0.21, 95% CI = −0.47 to 0.06).

#### 3.4.2 Effects of WBVT on ADL

**FIGURE 4:**
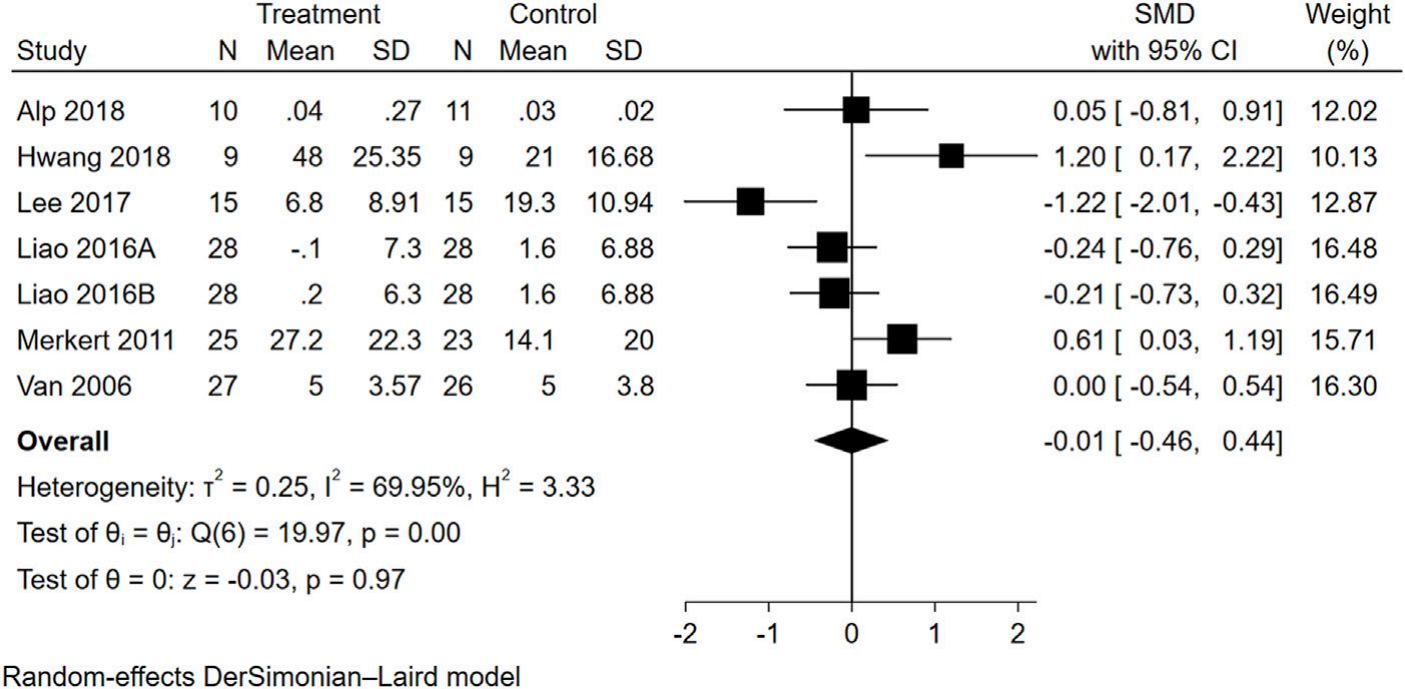
Forest plot showing the SMD and 95% CI of differences in ADL between the WBVT group and control group.

Seven trials from six studies reported data on ADL. As seen in Figure 4, the overall effect size of WBVT on ADL scale scores was insignificant (SMD = −0.01, 95% CI = −0.46 to 0.44, *p* = 0.97), suggesting insufficient evidence to determine the efficacy of WBVT on ADL. Subgroup analyses (Supplementary Figure S4) revealed that the effect sizes of the four ADL scales, namely, the BI (SMD = 0.29, 95% CI = −0.30 to 0.89), FAI (SMD = −0.22, 95% CI = −0.59 to 0.15), FIM (SMD = 0.05, 95% CI = −0.81 to 0.91), K-MBI (SMD = −0.03, 95% CI = −2.40 to 2.34), did not demonstrate statistical significance. The heterogeneity analysis of these studies was distinctly different (*I* ^2^ = 69.95%).

#### 3.4.3 Effects of WBVT on QOL

**FIGURE 5:**
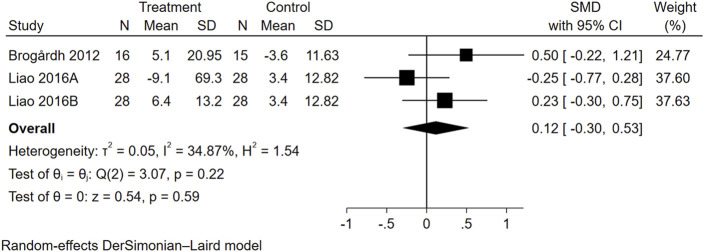
Forest plot showing the SMD and 95% CI of differences in QOL between the WBVT group and control group.

Three trials from two studies reported data on QOL (Figure 5). The overall effect size of WBVT on QOL scale scores was not significant (SMD = 0.12, 95% CI = −0.30 to 0.53).

### 3.5 Subgroup analysis

We conducted subgroup analysis based on the frequency mode and the vibration direction of WBVT. Regarding the parameters of WBVT, variable frequency vibration and side-alternating vibration exhibited significant efficacy in reducing spasticity, as well as improving motor and balance functions. Conversely, fixed frequency vibration and vertical vibration did not show significant therapeutic effects on any of these physical functions. (Table 2).

**TABLE 2:** Subgroup analysis of WBVT parameters on physical function.

Outcomes	WBVT parameters	SMD (95% CI)	*p*-values
Spasticity	Fixed frequency	−0.12 (−0.46 to 0.21)	*p* = 0.45
	Variable frequency	−0.61 (−1.00 to −0.22)	*p* < 0.01
	Side-alternating vibration	−0.65 (−1.07 to −0.23)	*p* < 0.01
	Vertical vibration	−0.14 (−0.46 to 0.17)	*p* = 0.34
Motor function	Fixed frequency	0.06 (−0.30 to 0.43)	*p* = 0.73
	Variable frequency	0.57 (0.30 to 0.85)	*p* < 0.01
	Side-alternating vibration	0.37 (0.12 to 0.62)	*p* < 0.01
	Vertical vibration	0.52 (−0.19 to 1.23)	*p* = 0.15
Balance function	Fixed frequency	0.18 (−0.05 to 0.40)	*p* = 0.13
	Variable frequency	0.46 (0.12 to 0.79)	*p* < 0.01
	Side-alternating vibration	0.27 (0.05 to 0.49)	*p* < 0.01
	Vertical vibration	0.28 (−0.06 to 0.63)	*p* = 0.11

Note: CI, confidence interval; SMD, standardized mean difference; WBVT, whole-body vibration training.

### 3.6 Publication bias and sensitivity analysis

In the physical function domain, including balance function and gait performance, the combined assessment of the funnel plot symmetry distribution (Supplementary Figure S5) and Egger’s test results (*p* = 0.0154 and *p* = 0.0556, respectively) suggested a possible publication bias. However, sensitivity analysis using the leave-one-out method did not show any significant changes in the overall conclusions, indicating the reliability of the results.

## 4 Discussion

The meta-analysis demonstrated that WBVT had a positive effect on reducing spasticity and improving motor and balance function, but its effects on gait, ADL, and QOL were inconclusive. Variable frequency vibration and side-alternating vibration showed significant efficacy in reducing spasticity and improving motor and balance functions, whereas fixed frequency vibration and vertical vibration did not yield significant therapeutic benefits in these domains.

### 4.1 Effects of WBVT on physical function

The underlying mechanism of WBVT remains unclear, but it is believed that vibration stimulation activates a variety of human skin and deep tissue receptors, especially class Ia and II afferent fibers, increasing sensory input to the central nervous system (Roll et al., 1989). This stimulation affects the excitability of functional brain areas such as the primary somatosensory cortex, primary motor areas, and premotor areas, promoting neuroplasticity (Murillo et al., 2014; Lapole et al., 2015; Lopez et al., 2017). Additionally, vibration stimulation on muscles can cause a tonic vibration reflex, leading to involuntary muscle contraction and affecting proprioception and motor performance (Murillo et al., 2014). Studies utilizing surface electromyography (sEMG) have demonstrated that WBVT can effectively activate muscles in individuals with conditions such as stroke and spinal cord injury (Dionello et al., 2017).

The results of our analysis demonstrated the anti-spasticity effect of WBVT, which was also confirmed in previous studies (Park et al., 2018; Yang et al., 2022). Since stroke patients may experience spasticity in various body parts (Wissel et al., 2010), and a recent meta-analysis has confirmed the effectiveness of WBVT in both upper and lower extremity spasticity (Zhang et al., 2023), it is essential to explore the effects of WBVT on spasticity in different body parts. However, most relevant trials did not report the specific spastic muscle groups assessed by MAS, making it difficult to determine the efficacy of WBVT for specific spastic muscles. Additionally, most included RCTs did not provide precise details regarding antispasmodic drug therapy, preventing us from further evaluating the possible role of these drugs in the anti-spasticity effect of WBVT. Future research should explore the effects of WBVT on spasticity in different body parts and the role of pharmacotherapy in the anti-spasticity effect of WBVT.

For motor function, previous meta-analyses mainly investigated the effect of WBVT on lower muscle strength, with a small effect size (Park et al., 2018; Yang and Butler, 2020). In contrast, our study aimed to directly explore the effect of WBVT on motor function using motor function scales. The meta-analysis revealed a significant effect size in favor of WBVT in improving motor function, particularly in the upper extremity. This result is consistent with a recent meta-analysis, although its credibility was limited due to language barriers (only two non-English reports were included) (Yang et al., 2022). However, variations in study designs prevented us from further analyzing the potential reasons for this conclusion. The significant effects observed on the FMA, indicating the positive impact of WBVT on motor function, highlight its potential as an effective intervention for stroke patients. Nonetheless, caution is advised when interpreting the non-significant effects found on the RMI and WMFT, considering the limited number of included studies. To validate these findings and explore the specific influence of WBVT on these motor function domains in stroke patients, future research with larger and more diverse samples is warranted. Furthermore, maintaining methodological consistency and using similar motor function scales in subsequent studies will facilitate accurate comparisons and enable integrated analyses.

Impaired balance and unstable posture increase the risk of falling in the majority of stroke patients, even with improved motor function. Poor balance control negatively affects the recovery of motor function and gait performance, which are crucial for restoring functional independence in stroke patients (de Haart et al., 2004; Lee et al., 2016b). Our meta-analysis supports previous findings that WBVT improvs balance scores in patients with stroke (Yang and Butler, 2020; Yin et al., 2022). The possible mechanisms of balance recovery after WBVT may be an increase in proprioception and improvement in weight-bearing to the hemiplegic side (Hwang, 2018). However, the impact of publication bias in the included studies should be considered when interpreting our results, although sensitivity analysis indicated that its impact may be limited. Therefore, further studies with larger sample sizes, standardized intervention protocols, and consistent outcome measures are needed to confirm our findings.

To gain a deeper understanding of the varying effects of WBVT on different balance assessment scales, we conducted a subgroup analysis specifically on the balance scale. Our findings indicate a significant therapeutic effect of WBVT in improving balance function in stroke patients when utilizing the BBS. This may be attributed to the high sensitivity and accuracy of the BBS in assessing balance function in this population. However, it is worth noting that the other five balance scales (ABC, K-BBS, MFRT, Mini-BESTest, and TPOMA) did not show significant effect sizes. The limited number of studies available for analysis and the potential influence of multiple factors on the results of a single study should be considered, which may impact the reliability of these conclusions. Therefore, additional studies are necessary to strengthen support for these findings. In conclusion, while WBVT has demonstrated positive effects in improving balance function in stroke patients, its applicability may vary across different balance assessment scales. Further research is needed to validate and substantiate these findings.

For gait performance, various clinical scales are used to evaluate the improvement in gait performance of individuals with stroke. These include the 6MWT to assess walking endurance (Fulk et al., 2008), the FAC with 6 evaluation levels of gait capacity (Mehrholz et al., 2007), the TPOMA with 7 gait evaluation items (such as starting, step length, gait symmetry, step continuity, walking path, and step width) (Abbruzzese, 1998), the TUGT to assess balance capability and functional mobility (Podsiadlo et al., 1991), and the 10MWT to reflect changes in walking speed (Holden et al., 1984). Our study demonstrated a small but significant effect size of the 10MWT, suggesting a limited but positive effect of WBVT on walking speed. The potential benefits of WBVT on walking speed might include the reduction of ankle spasticity and improvement in ankle joint control (Goldie et al., 2001; Lamontagne et al., 2002). However, our meta-analysis showed an insignificant effect size for the total random effect of gait scale scores, including the 6MWT, FAC, TPOMA, and TUGT. These results suggest that WBVT did not improve overall gait performance compared to controls, which is inconsistent with previous meta-analyses (Yang and Butler, 2020; Yin et al., 2022). The difference in results may be attributed to the mixed study design due to differences in inclusion exclusion criteria, study methodology, and other factors across studies.

Although clinical scales are commonly used to evaluate gait performance in patients with stroke, they lack objective and accurate indicators to reflect temporospatial variables of gait, such as step length, cadence, and walking speed. Previous studies have reported mixed results regarding the effects of WBVT on gait variables in stroke patients. Some studies have shown significant improvements in walking speed, step length, and limb support (Choi et al., 2017; Sade et al., 2020), while others have not observed significant differences in gait variables between the WBVT group and control group (Lee, 2019). A recent meta-analysis also preliminarily validated the significant improvement of WBVT in terms of stride length, walking speed, cadence and stride length in stroke patients (Yin et al., 2022). However, differences in measurement devices may lead to measurement biases that could affect the results of the analysis. Due to the limited number of RCTs using computerized gait analysis, more research is needed to draw reliable conclusions on the effectiveness of WBVT in improving gait in stroke patients.

### 4.2 Effects of WBVT on ADL and QOL

The level of independence in performing ADL is crucial for the QOL of stroke patients (Clarke et al., 2000; Kim et al., 2014). Therefore, when developing rehabilitation programs, it is essential to address not only physical function improvement but also the reduction of ADL dependency and enhancement of QOL in an integrated manner. Unlike previous meta-analyses, our comprehensive study aimed to analyze the rehabilitative effects of WBVT in stroke patients, specifically examining its impact on ADL and QOL. However, our analysis of ADL scores revealed a non-significant overall effect size of WBVT, indicating insufficient evidence to determine its efficacy in improving ADL. It is important to acknowledge the considerable heterogeneity among the included studies, which may be attributed to variations in study design, sample characteristics, vibration treatment protocols, and ADL assessment tools employed, leading to inconsistency and uncertainty in the study results.

Interestingly, a study by Hwang (Hwang, 2018) reported improvements in walking ability and ADL in stroke patients who received WBVT, particularly among those showing enhanced balance scores. This finding suggests that enhancing balance has a significant impact on improving gait performance and ADL. Our findings indicate that WBVT may be effective in improving spasticity, motor function, and balance in stroke patients. However, it is important to note that positive outcomes in physical function do not always translate into improvements in ADL, as observed in some studies (Hwang, 2018). Another important consideration is that the recovery of walking ability is crucial for improving ADL and QOL in stroke patients (Xu et al., 2016; Wonsetler and Bowden, 2017), which may lead to conflicting outcomes. Therefore, future research on WBVT in stroke patients should focus on standardizing protocols, considering sample characteristics, utilizing validated outcome measures, improving study design, and investigating underlying mechanisms to enhance understanding of its effects on ADL.

Impaired physical function and mobility have consistently been linked to decreased QOL in stroke patients (Clarke et al., 2000; Geyh et al., 2007; Teixeira-Salmela et al., 2009; Gunaydin et al., 2011). Recognizing the importance of QOL, we aimed to assess the effectiveness of WBVT on QOL outcomes. However, the limited number of available RCTs in this specific area contributes to the uncertainty of our study’s findings. Future research should prioritize investigating the impact of WBVT on QOL to provide a more comprehensive understanding of its potential benefits in stroke patients.

### 4.3 Moderators of WBVT effects

The optimal parameters of WBVT for functional recovery in stroke patients remains uncertain. Our subgroup analysis showed that variable frequency and side-alternating vibration had a more significant therapeutic effect on reducing spasticity, improving motor and balance function, while evidence for fixed frequency and vertical vibration was lacking. Thus, variable frequency and side-alternating vibration of WBVT differentiated more effective and less effective trials, providing valuable information for designing future WBVT-based trial protocols. Perchthaler et al. (Perchthaler et al., 2015) also suggested that using side-alternating vibration and increasing the knee flexion position could reduce head acceleration and damage to the eyes and ears. However, it is worth noting that the WBVT used in the included trials was mostly individually prescribed by professionals to minimize the risk of potential injuries, such as falling during training. Therefore, when implementing precise, individualized WBVT in clinical practice, modulating factors of successful interventions should be taken into account. Distinguishing the characteristics of successful and unsuccessful interventions is crucial in optimizing the therapeutic benefits of WBVT for stroke patients.

### 4.4 Limitations of the meta-analysis

This meta-analysis had several limitations. Firstly, the analysis did not include an examination of other parameters of WBVT, such as amplitude and stimulation time. This omission was due to the variations in vibration equipment and training programs across the included studies. Therefore, following comprehensive guidelines for reporting WBVT studies will help to improve the quality and comparability of future WBVT studies in stroke, thereby promoting the development and deepening of the clinical application of WBVT in stroke (Wuestefeld et al., 2020). Secondly, the limited number of RCTs assessing QOL outcomes restricted our ability to comprehensively evaluate the effectiveness of WBVT in this domain. Furthermore, the utilization of different clinical function scales among the included studies may have introduced measurement heterogeneity, despite efforts to minimize its impact through the calculation of SMD values. Moreover, the wide confidence intervals and the quality of the studies included in this meta-analysis may limit the generalizability and interpretation of the results. Therefore, further well-designed studies are warranted to validate and confirm the findings presented in this meta-analysis.

## 5 Conclusion

In summary, based on the findings of this meta-analysis, WBVT shows potential in relieving spasticity and enhancing motor and balance functions in stroke patients. Subgroup analysis indicates that variable frequency and side-alternating vibration of WBVT may play a significant role in achieving therapeutic effects for these specific dysfunctions. However, further research is necessary to investigate the efficacy of WBVT on gait, ADL, and QOL in patients with stroke. The findings of this study contribute to the development of individualized and precise WBVT protocols for stroke rehabilitation. Nonetheless, additional studies are necessary to validate and expand upon these findings.

## Data Availability

The original contributions presented in the study are included in the article/Supplementary Material, further inquiries can be directed to the corresponding author.

## Supplementary material

SUPPLEMENTARY TABLE S1 Search strategies for different databases.

SUPPLEMENTARY FIGURE S1 Subgroup analysis results for motor function scales.

SUPPLEMENTARY FIGURE S2 Subgroup analysis results for balance function scales.

SUPPLEMENTARY FIGURE S3 Subgroup analysis results for gait scales.

SUPPLEMENTARY FIGURE S4 Subgroup analysis results for ADL scales.

SUPPLEMENTARY FIGURE S5 Publication bias funnel plots. **(A)** Balance function; **(B)** Gait.
