# Supplementary material for: Effects of whole-body vibration training on physical function, activities of daily living, and quality of life in patients with stroke: a systematic review and meta-analysis
Source: Front Physiol. 2024 Jan 23;15:1295776. doi: 10.3389/fphys.2024.1295776 (PMC10844406; doi:10.3389/fphys.2024.1295776)
Supplement: Supplementary file 1 [file DataSheet1.zip › Datasheet 1/SUPPLEMENTARY TABLE S1.docx]

**Supplementary Table S1** Search strategies for different databases.

| **Database** | **#** | **Query** |
| --- | --- | --- |
| PubMed | 1 | "cerebrovascular disorders"[MeSH Terms] |
|  | 2 | "brain injuries"[MeSH Terms] |
|  | 3 | "brain damage, chronic"[MeSH Terms] |
|  | 4 | "hemiplegia"[MeSH Terms] |
|  | 5 | "paresis"[MeSH Terms] |
|  | 6 | "gait disorders, neurologic"[MeSH Terms] |
|  | 7 | "stroke*"[Title/Abstract] OR "cva"[Title/Abstract] OR "poststroke"[Title/Abstract] OR "post-stroke"[Title/Abstract] |
|  | 8 | "cerebrovasc*"[Title/Abstract] OR "cerebral vascular"[Title/Abstract] |
|  | 9 | "cerebral"[Title/Abstract] OR "cerebellar"[Title/Abstract] OR "brain*"[Title/Abstract] OR "vertebrobasilar"[Title/Abstract] |
|  | 10 | "infarct*"[Title/Abstract] OR "ischaemi*"[Title/Abstract] OR "ischemi*"[Title/Abstract] OR "thrombo*"[Title/Abstract] OR "emboli*"[Title/Abstract] OR "apoplexy"[Title/Abstract] |
|  | 11 | #9 and #10 |
|  | 12 | "cerebral"[Title/Abstract] OR "brain"[Title/Abstract] OR "subarachnoid"[Title/Abstract] |
|  | 13 | "haemorrhage"[Title/Abstract] OR "hemorrhage"[Title/Abstract] OR "haematoma"[Title/Abstract] OR "hematoma"[Title/Abstract] OR "bleed*"[Title/Abstract] |
|  | 14 | #12 and #13 |
|  | 15 | "hemipleg*"[Title/Abstract] OR "paresis"[Title/Abstract] OR "paretic"[Title/Abstract] OR "brain injur*"[Title/Abstract] |
|  | 16 | #1 or #2 or #3 or #4 or #5 or #6 or #7 or #8 or #11 or #14 or #15 |
|  | 17 | "vibration"[MeSH Terms] |
|  | 18 | "vibration*"[Title/Abstract] OR "whole body vibration"[Title/Abstract] |
|  | 19 | #17 or #18 |
|  | 20 | (randomized controlled trial[pt] OR controlled clinical trial[pt] OR randomized[tiab] OR placebo[tiab] OR clinical trials as topic[mesh:noexp] OR randomly[tiab] OR trial[ti]) NOT (animals [mh] NOT (humans [mh] AND animals[mh])) |
|  | 21 | #16 and #19 and #20 |
| Cochrane Library | 1 | MeSH descriptor: [Cerebrovascular Disorders] explode all trees |
|  | 2 | MeSH descriptor: [Brain Injuries] explode all trees |
|  | 3 | MeSH descriptor: [Brain Damage, Chronic] explode all trees |
|  | 4 | MeSH descriptor: [Hemiplegia] explode all trees |
|  | 5 | MeSH descriptor: [Paresis] explode all trees |

**Supplementary Table S1 (Continued)** Search strategies for different databases.

| **Database** | **#** | **Query** |
| --- | --- | --- |
|  | 6 | MeSH descriptor: [Gait Disorders, Neurologic] explode all trees |
|  | 7 | (stroke* or cva or poststroke or post‐stroke or cerebrovasc* or cerebral vascular):ti,ab,kw |
|  | 8 | (cerebral or cerebellar or brain* or vertebrobasilar):ti,ab,kw |
|  | 9 | (infarct* or ischaemi* or ischemi* or thrombo* or emboli* or apoplexy):ti,ab,kw |
|  | 10 | #8 and #9 |
|  | 11 | (cerebral or brain or subarachnoid):ti,ab,kw |
|  | 12 | (haemorrhage or hemorrhage or haematoma or hematoma or bleed*):ti,ab,kw |
|  | 13 | #11 and #12 |
|  | 14 | (hemipleg* or hemipar* or paresis or paretic or brain injur*):ti,ab,kw |
|  | 15 | #1 or #2 or #3 or #4 or #5 or #6 or #7 or #10 or #13 or #14 |
|  | 16 | MeSH descriptor: [Vibration] explode all trees |
|  | 17 | (vibration* or whole body vibration):ti,ab,kw |
|  | 18 | #16 or #17 |
|  | 19 | #15 and #18 |
| Embase | 1 | 'cerebrovascular disease'/exp |
|  | 2 | 'brain injury'/exp |
|  | 3 | 'chronic brain disease'/exp |
|  | 4 | 'hemiplegia'/exp |
|  | 5 | 'paresis'/exp |
|  | 6 | 'neurologic gait disorder'/exp |
|  | 7 | stroke*:ab,ti OR cva:ab,ti OR poststroke:ab,ti OR post‐stroke:ab,ti OR cerebrovasc*:ab,ti OR 'cerebral vascular':ab,ti |
|  | 8 | cerebral:ab,ti OR cerebellar:ab,ti OR brain*:ab,ti OR vertebrobasilar:ab,ti |
|  | 9 | infarct*:ab,ti OR ischaemi*:ab,ti OR ischemi*:ab,ti OR thrombo*:ab,ti OR emboli*:ab,ti OR apoplexy:ab,ti |
|  | 10 | #8 AND #9 |
|  | 11 | cerebral:ab,ti OR brain:ab,ti OR subarachnoid:ab,ti |
|  | 12 | haemorrhage:ab,ti OR hemorrhage:ab,ti OR haematoma:ab,ti OR hematoma:ab,ti OR bleed*:ab,ti |
|  | 13 | #11 AND #11 |
|  | 14 | hemipleg*:ab,ti OR paresis:ab,ti OR paretic:ab,ti OR 'brain injur*':ab,ti |
|  | 15 | #1 OR #2 OR #3 OR #4 OR #5 OR #6 OR #7 OR #10 OR #13 OR #14 |
|  | 16 | 'vibration'/exp |
|  | 17 | vibration*:ab,ti OR 'whole body vibration':ab,ti |
|  | 18 | #17 OR #18 |

**Supplementary Table S1 (Continued)** Search strategies for different databases.

| **Database** | **#** | **Query** |
| --- | --- | --- |
|  | 19 | 'crossover procedure':de OR 'double-blind procedure':de OR 'randomized controlled trial':de OR 'single-blind procedure':de OR random*:de,ab,ti OR factorial*:de,ab,ti OR crossover*:de,ab,ti OR ((cross NEXT/1 over*):de,ab,ti) OR placebo*:de,ab,ti OR ((doubl* NEAR/1 blind*):de,ab,ti) OR ((singl* NEAR/1 blind*):de,ab,ti) OR assign*:de,ab,ti OR allocat*:de,ab,ti OR volunteer*:de,ab,ti |
|  | 20 | #15 AND #18 AND #19 |
