# Supplementary figures and images for: Effects of whole-body vibration training on physical function, activities of daily living, and quality of life in patients with stroke: a systematic review and meta-analysis
Source: Front Physiol. 2024 Jan 23;15:1295776. doi: 10.3389/fphys.2024.1295776 (PMC10844406; doi:10.3389/fphys.2024.1295776)

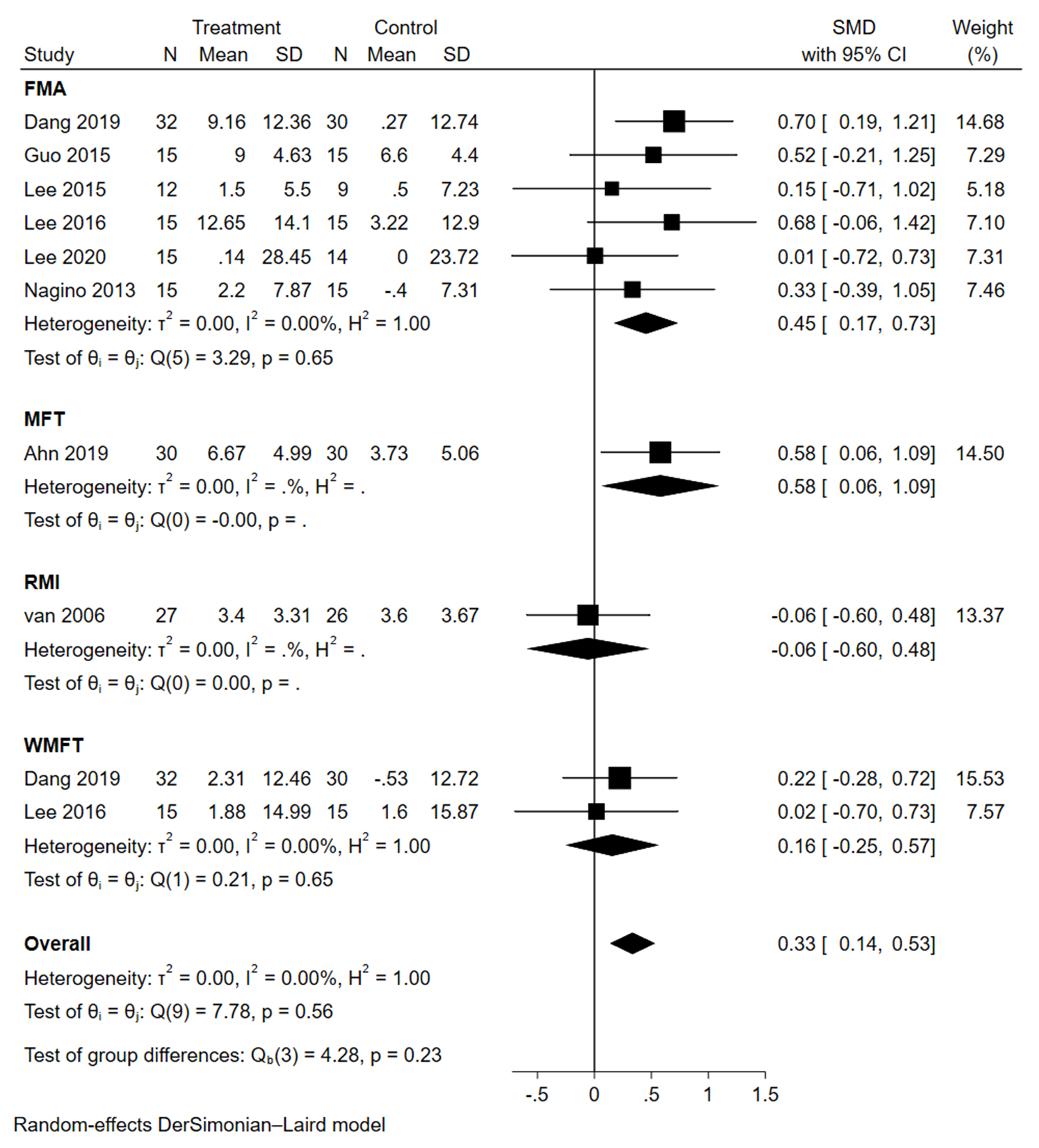

Supplement: Supplementary file 1 [file DataSheet1.zip › Datasheet 1/SUPPLEMENTARY FIGURE S1.tif]

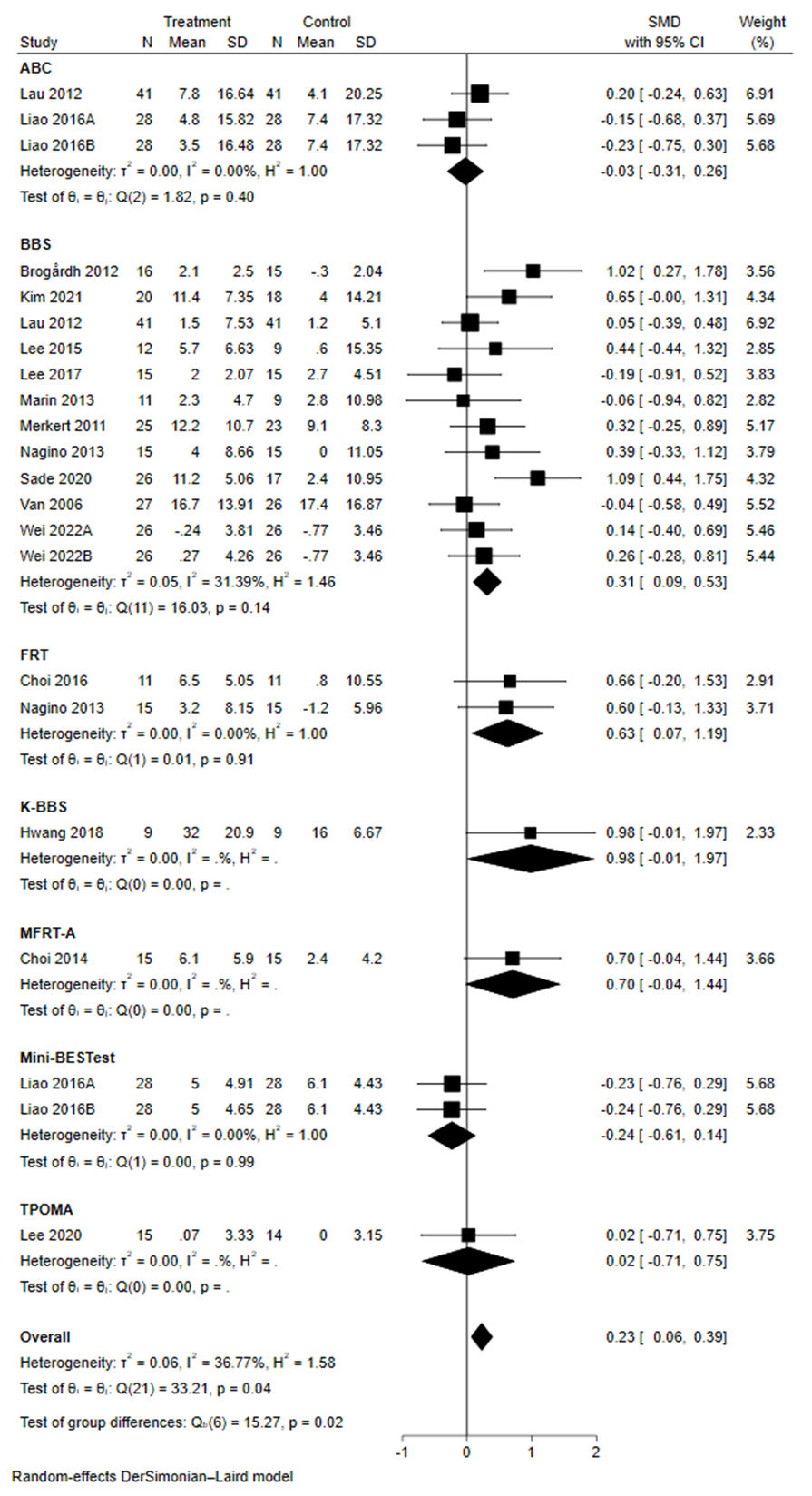

Supplement: Supplementary file 1 [file DataSheet1.zip › Datasheet 1/SUPPLEMENTARY FIGURE S2.tif]

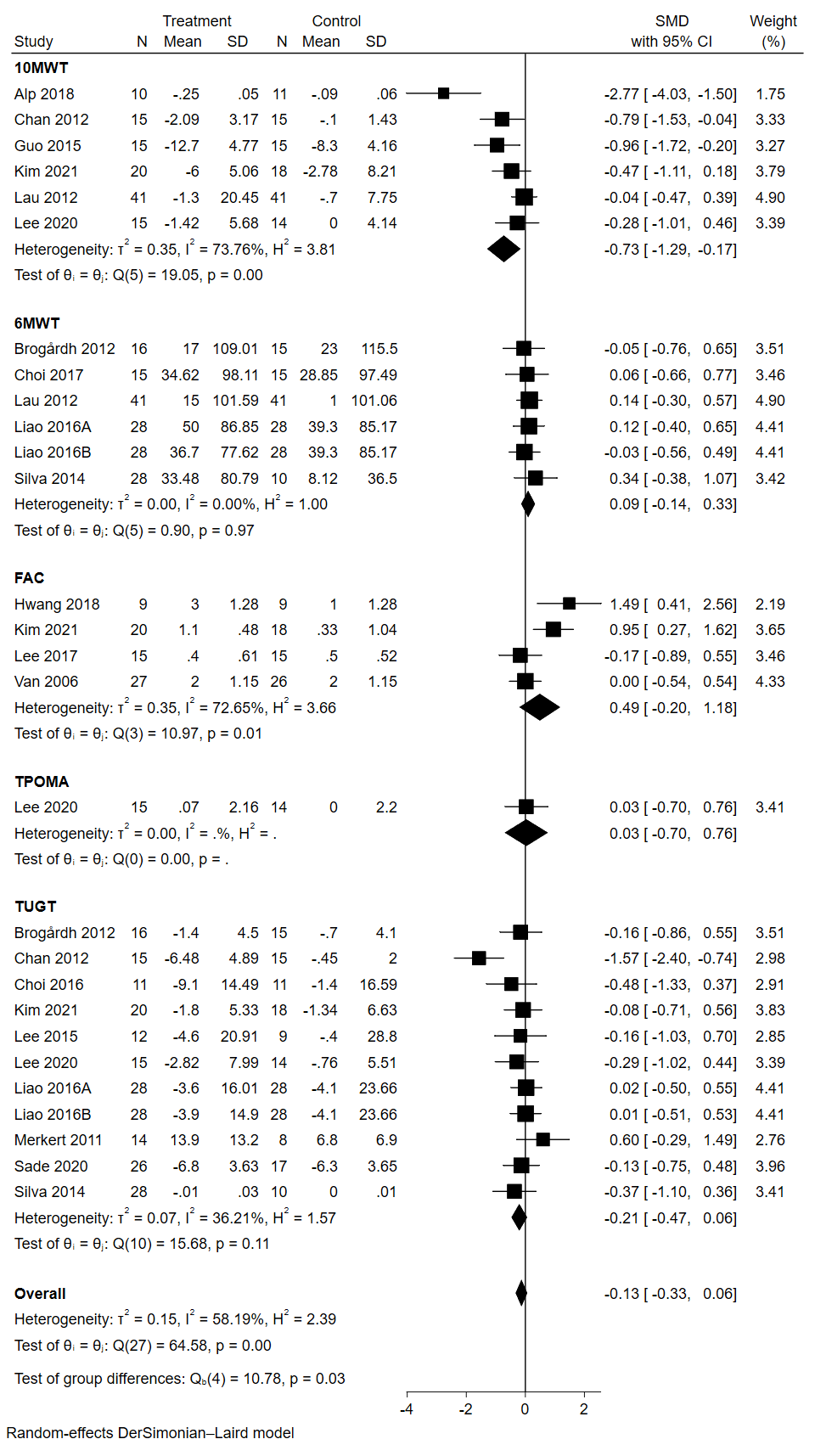

Supplement: Supplementary file 1 [file DataSheet1.zip › Datasheet 1/SUPPLEMENTARY FIGURE S3.tif]

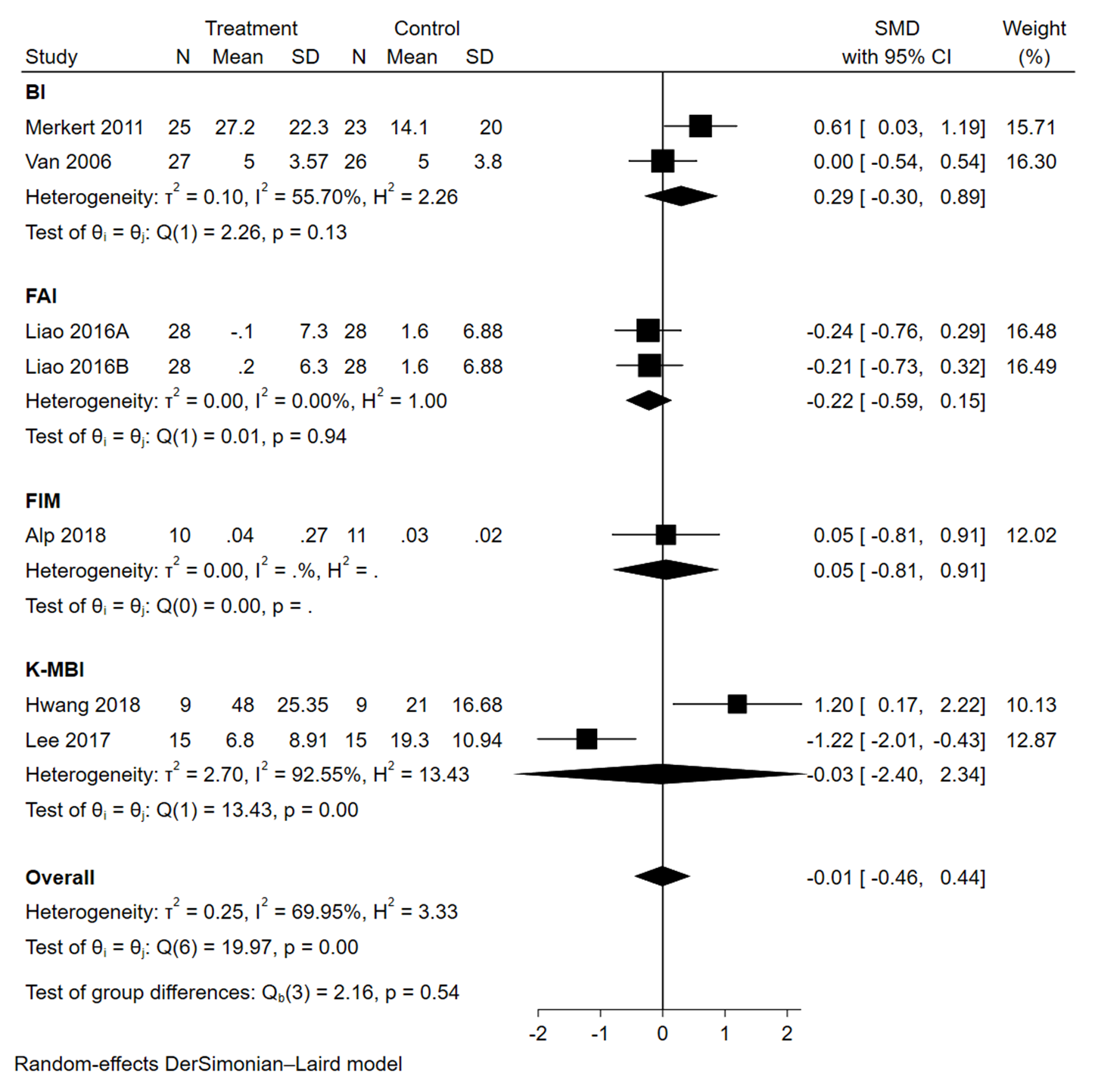

Supplement: Supplementary file 1 [file DataSheet1.zip › Datasheet 1/SUPPLEMENTARY FIGURE S4.tif]

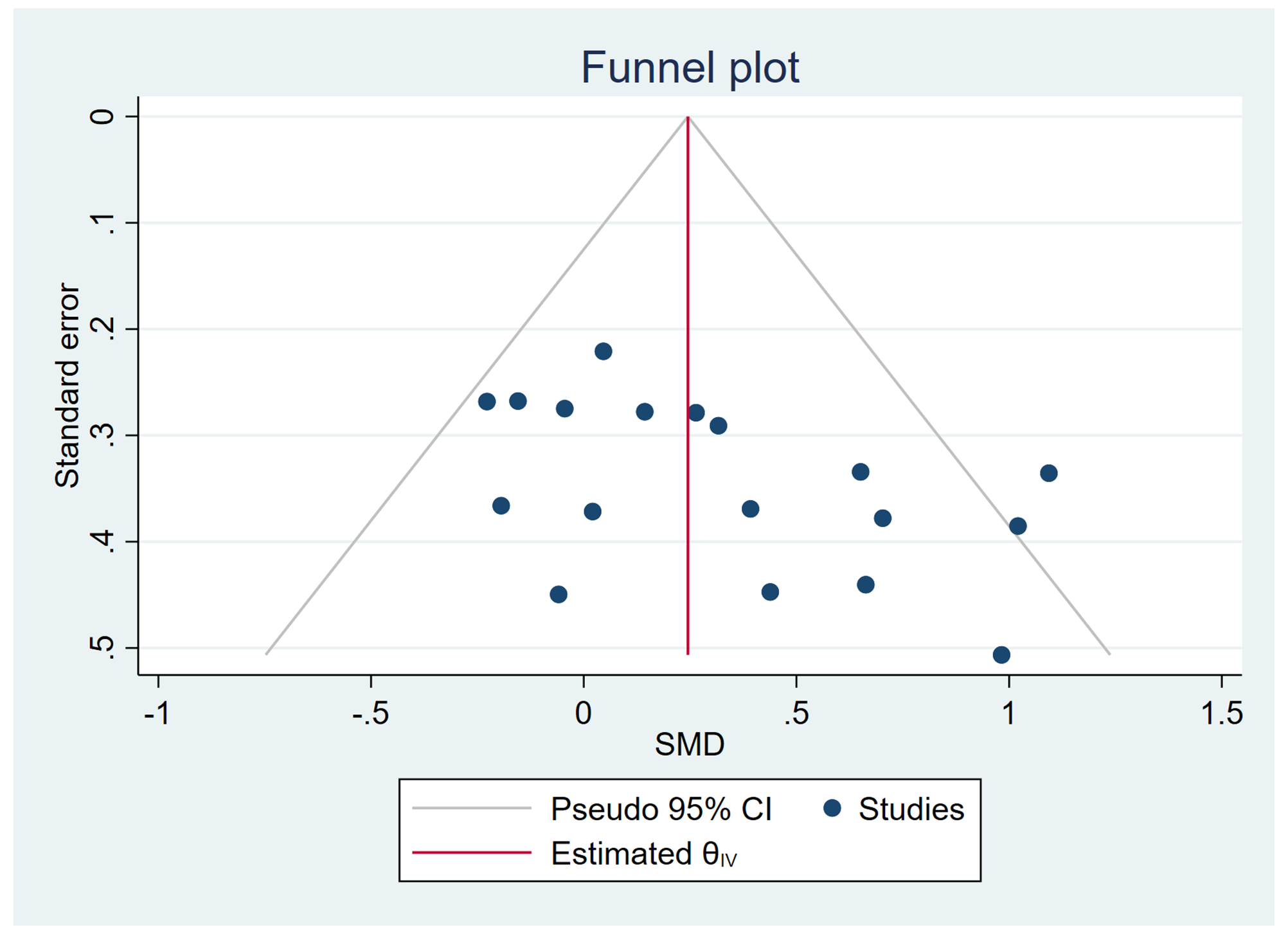

Supplement: Supplementary file 1 [file DataSheet1.zip › Datasheet 1/SUPPLEMENTARY FIGURE S5A.tif]

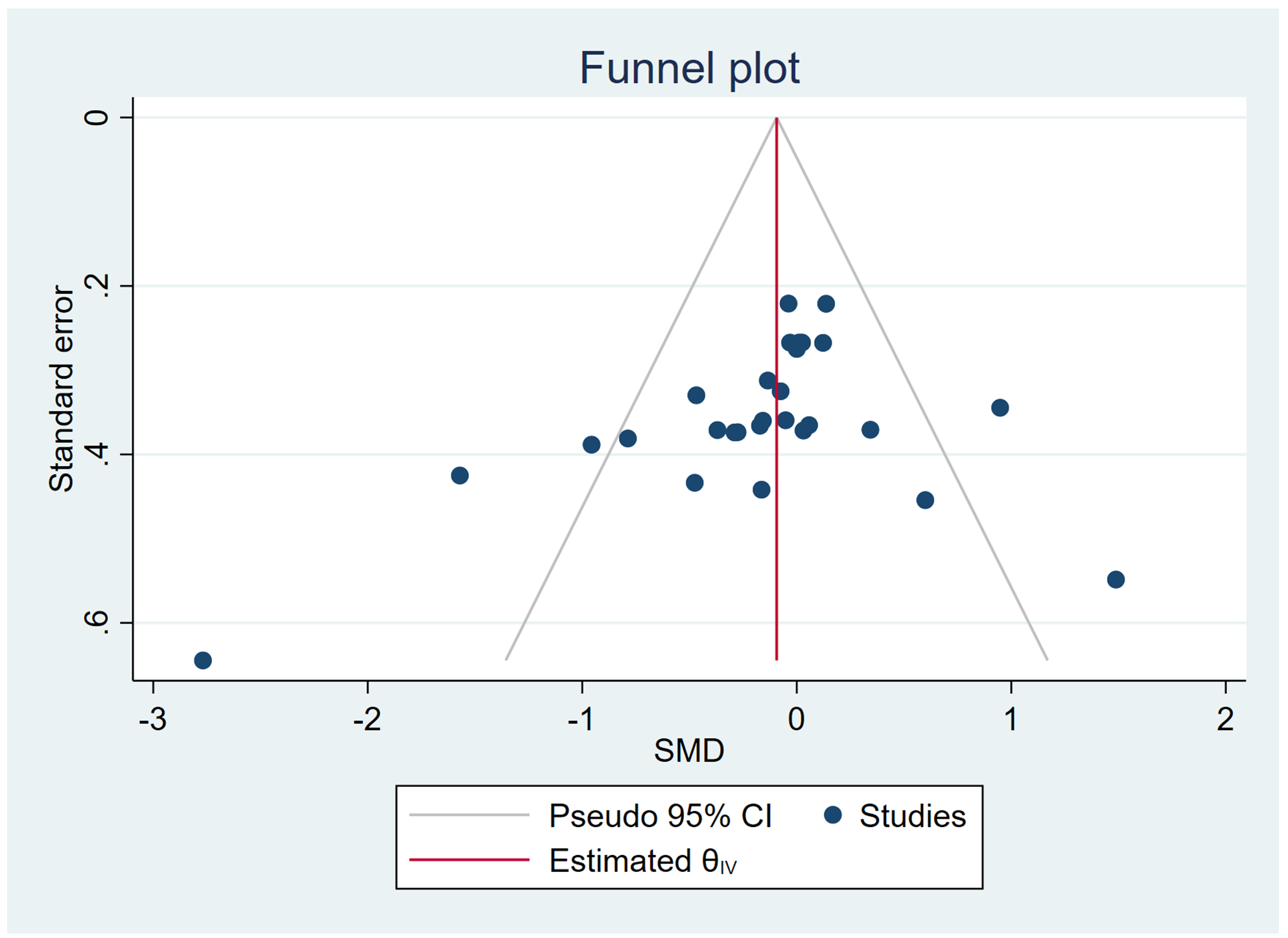

Supplement: Supplementary file 1 [file DataSheet1.zip › Datasheet 1/SUPPLEMENTARY FIGURE S5B.tif]
